# Effect of Ca–Al–Si–O common glass on dielectric properties of low-temperature co-fired ceramic materials with different fillers

**DOI:** 10.1080/13102818.2014.949039

**Published:** 2014-10-21

**Authors:** Zee-hoon Park, Dong-hun Yeo, Hyo-soon Shin

**Affiliations:** ^a^ R & D Center, SCC Co., Ltd., Gumi, Gyeongsangbuk-do, Korea; ^b^ Engineering Ceramic Team, Korea Institute of Ceramic Engineering and Technology, Icheon, Gyeonggi, Korea

**Keywords:** LTCC, common glass, low loss, filler, Ca–Al–Si–O glass

## Abstract

High-density integration in single component used for mobile communication is highly demanded with the miniaturization trend in multi-functional light-weighted mobile communication devices. Embedding passive components into multi-layered ceramic chips is also increasingly needed for high integrity. The need for high strength materials to be used in handheld devices has also increased. To this end, many attempts to join different low-temperature co-fired ceramics (LTCC) materials with different dielectric constants have been made, but failed with de-laminations or internal cracks mainly due to difference of thermal expansion coefficients. It is thought that this difference could be minimized with the use of common glass in different LTCC materials. In this study, several candidates of common glass were mixed with various fillers of LTCC to have various dielectric constants in the radio-frequency, and to minimize the mismatch in joining. Ca–Al–Si–O glass was mixed with 1.3MgO-TiO_2_, cordierite and CaTiO_3_. Mixtures were tape-cast and sintered to be compared with their micro-structures, dielectric properties and thermo-mechanical characteristics. When 1.3MgO-TiO_2_ with volumetric ratio of 30% was mixed with Ca–Al–Si–O glass, the measured dielectric constant was 7.9, the quality factor was 3708. With 45 volumetric percent of cordierite, the dielectric constant was 5 and the quality factor was 1052.

## Introduction

**Figure 1.**
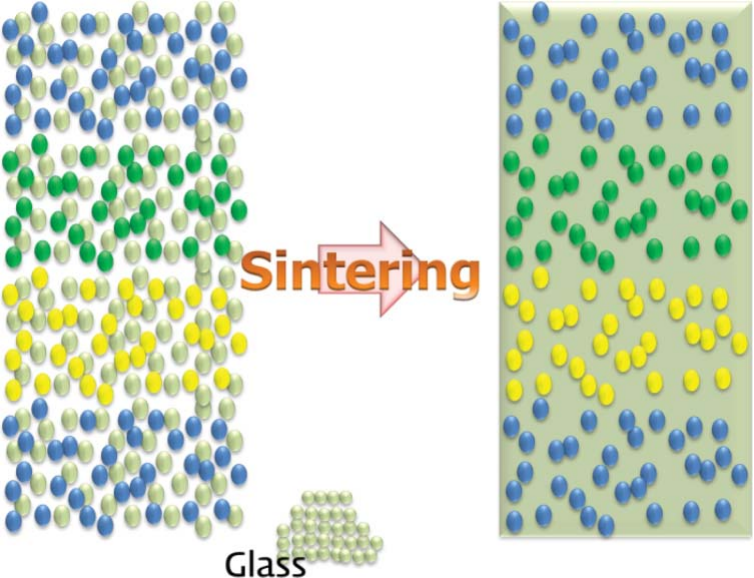
Schematic diagram of common glass for LTCC.

Material demand in radio-frequency communication technology has led to the development of low-temperature co-fired ceramic (LTCC) materials which have dielectric constants less than 10. For the purpose of achieving high integrity, recent attempts have focused on embedding of passive components into LTCC modules. Some passive components, e.g. capacitors that need a very high capacitance, could not be made by LTCC with a limited thickness of substrate. While a lot of efforts have been put into the development of materials that are sinterable at low temperature, fewer studies seem to focus on the problems associated with junctions between different materials. On the one hand, shrinkage differences between different materials result in warpage and delamination and, on the other hand, a chemical reaction may occur at the sintering temperature. It is important to find a solution to these two main problems.[1–4] In this study, in order to suppress the coefficient of thermal expansion (CTE) difference among materials, different LTCC materials which have different fillers with common glass for different dielectric properties were devised as shown in Figure 1. ‘Filler with glass’ is the most common form of LTCC materials. Efforts have been made to co-fire these kinds of ‘fillers with common glass’ without cracking or de-lamination.[5]

## Materials and methods

**Figure 2.**
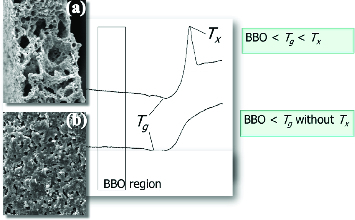
Requirement on LTCC glass: incomplete densification (a) vs. full densification (b).

The requirements on LTCC glass are shown in Figure 2. When glass crystallizes at a temperature below the sintering temperature, though the glass transition temperature of the chosen glass is higher than that of binder decomposition, the crystallized glass forms necks among fillers not to perform full densification as shown in Figure 2(a). However, if the chosen glass does not crystallize below the sintering temperature and has a higher transition temperature than that of binder decomposition, the glass with sufficiently low viscosity could fill the space among filler particles as shown in Figure 2(b). Low viscosity glass (SGL, SCC Co., Ltd., KOREA) was chosen as common glass. Its glass (transition) temperature is about 700 °C, and it does not crystallize. The glass does not react with candidate fillers in this study during firing. Various fillers were considered such as 1.3MgO-TiO_2_, 2La_2_O_3_-TiO_2_, cordierite and CaTiO_3_. All filler powders were prepared by Cepotech Co., Ltd., (Korea). Fillers were weighed with a volumetric ratio of 20, 25, 30, 35, 40 and 45 to glass. Weighed filler and glass were milled with a ball mill for 24 h for mixing, and then organic additives such as binder and plasticizer were added and mixed for 24 h with a ball mill. Prepared slurries were cast into green sheets and laminated. Laminated specimens were sintered at 850 °C for 30 min. The dielectric properties of sintered specimens were measured by a network analyser (8720ES, Agilent, USA), and their structure was observed by field emission scanning electron microscopy (FE-SEM, LSM-6700F, JEOL, Japan).

## Results and discussion

When two different dielectric materials are mixed, it is known that the relative dielectric constant of the mixture is expressed as shown in the following equation (known as Maxwell's relation) with the volumetric ratio of each material:


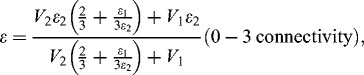


where *ϵ* is the relative dielectric constant of the mixture, *V*_1_ is the volumetric ratio of the material ‘1’, *ϵ*_1_ is the relative dielectric constant of the material ‘1’, *V*_2_ is the volumetric ratio of the material ‘2’ and *ϵ*_2_ is the relative dielectric constant of the material ‘2’.

**Figure 3.**
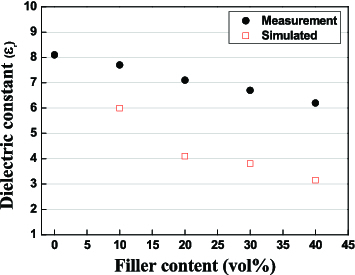
Dielectric constant variation with silica filler contents in LTCC.

In this study, different volumetric ratios of the filler (from 10% to 40%) were used, and the predicted dielectric constants and the measured ones (at 1 MHz) are compared in Figure 3. The dielectric constant of glass was 8.1 and that of the chosen filler (silica) was 4. In the figure, the predicted values were varied from 8.1 to 6.2 linearly, but the measured ones are relatively lower than the predicted ones. This phenomenon is thought to be caused by the increased pore amount formed around filler particles during sintering. In other words, it is expected that there are only two phases of glass and filler, but as the filler amount increases, that of pores, which has a very low dielectric constant of 1, also increases, and then the real dielectric value is thought to appear relatively low. The dielectric constant of the mixture does not accord with Maxwell's relation for only two phases.

Tables 1–3 show the sintered densities, the dielectric constants and the quality factors measured with various filler contents and different heating rates during sintering. Fillers were 1.3MgO-TiO_2_ and cordierite. Moreover, the specimens were sintered at 850 °C for 30 minutes. The heating rate was varied: 2, 5 and 10 °C/min. With the 1.3MgO-TiO_2_ filler, the highest sintered densities were obtained at a volumetric ratio of 30%, as 3.02–3.04 g/cm^3^. With cordierite, the highest ones were obtained at a volumetric ratio of 40% as 2.44–2.59 g/cm^3^. With 1.3MgO-TiO_2_, there was no remarkable difference between the densities with the heating rate, but with cordierite, the sintered density at a heating rate of 2 °C/min was remarkably lower than those at 5 and 10 °C/min. It is thought that at a low heating rate of 2 °C/min, the viscosity of the melted glass was too low to infiltrate into the filler particles, and therefore the densification during the sintering process was insufficient.

**Table 1.** Sintered densities with various filler contents and different heating rate during sintering.

	1.3MgO-TiO_2_			Cordierite		
Content (vol%)	2 °C/min	5 °C/min	10 °C/min	2 °C/min	5 °C/min	10 °C/min
25	2.96	2.99	2.98			
30	3.03	3.02	3.04			
35	2.91	2.99	2.99	2.39	2.59	2.54
40				2.44	2.59	2.55
45				2.38	2.58	2.54

**Table 2.** Dielectric constants with various filler contents and different heating rate during sintering.

	1.3MgO-TiO_2_			Cordierite		
Content (vol%)	2 °C/min	5 °C/min	10 °C/min	2 °C/min	5 °C/min	10 °C/min
25	8.9	8.4	8.3			
30	10.6	9.4	7.9			
35	9.3	8.6	9.2	5.9	5.5	5.1
40				4.6	5.1	5.1
45				4.8	5.2	5

**Table 3.** Quality factors with various filler contents and different heating rate during sintering.

	1.3MgO-TiO_2_			Cordierite		
Content (vol%)	2 °C/min	5 °C/min	10 °C/min	2 °C/min	5 °C/min	10 °C/min
25	779	760	1145			
30	1273	2719	3708			
35	1232	1674	1135	357	821	862
40				1010	1021	672
45				982	913	1052

As shown in Table 2, with 1.3MgO-TiO_2_, the dielectric constants ranged from 7.9 to 10.6, while with cordierite, from 4.6 to 5.9. This is thought to be caused by the relatively higher dielectric constant of 1.3MgO-TiO_2_ than that of cordierite (about 5). MgO and TiO_2_ are known as typical dielectric materials. As shown in Table 3, the relatively high values of the quality factor above 1000 were obtained in both cases of 1.3MgO-TiO_2_ and cordierite. Especially, when 1.3MgO-TiO_2_ at a volumetric ratio of 30% was mixed with glass and then sintered at a heating rate of 10°C/min, the highest quality factor of 3708 was obtained. With cordierite contents of 40 and 50 volumetric%, high-quality factors above 900 were obtained.

**Figure 4.**
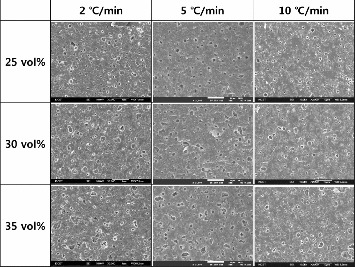
Sintered microstructures of LTCC with 1.3MgO-TiO_2_ content variation and different heating rates.

**Figure 5.**
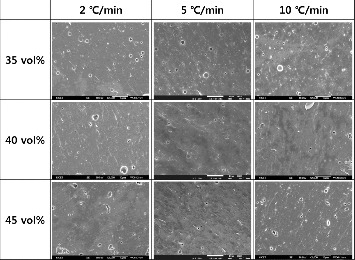
Sintered microstructures of LTCC with cordierite content variation and different heating rates.

Figures 4 and 5 show the sintered microstructures of LTCCs with different filler contents and different heating rates. Figure 4 is for 1.3MgO-TiO_2_, and Figure 5 for cordierite. Specimens were sintered at 850 °C for 30 min. With 1.3MgO-TiO_2_, relatively many pores were observed, but the pore content decreased with increasing the heating rate. As mentioned above, it is thought that at a high heating rate, the lower viscosity of melted glass could better the space around filler particles.[6] With cordierite, there were relatively less pores observed than in 1.3MgO-TiO_2_. At a heating rate of 5 °C/min and filler content of 40%, the least pores were observed and this accords with the quality factor result.

**Figure 6.**
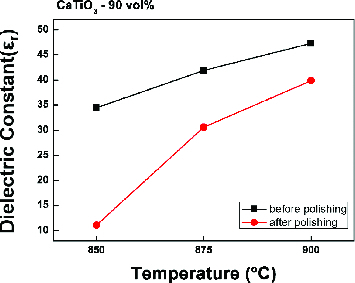
Dielectric constant before and after polishing.

Figure 6 shows the measured dielectric constants when CaTiO_3_ (90 volumetric%) was mixed with glass and then sintered at 850, 875 and 900 °C. The dielectric constants were measured twice before and after polishing. With increase of the sintering temperature, the measured dielectric constant also increased. As CaTiO_3_ has a relatively high dielectric constant,[7] the highest measured value of LTCC was 47 at 900 °C. It was expected that with polishing of the sintered specimen, the measured dielectric constant would become higher, as roughness on the surface was removed. However, the measured dielectric constants after polishing were remarkably lower than those before polishing.

The surface microstructures before and after polishing were compared and there was a dense glass region near the surfaces and a relatively vacant region in the deeper parts of the specimens. With polishing, the dense glass region was removed, and open pores appeared to lower the dielectric constants.

**Figure 7.**
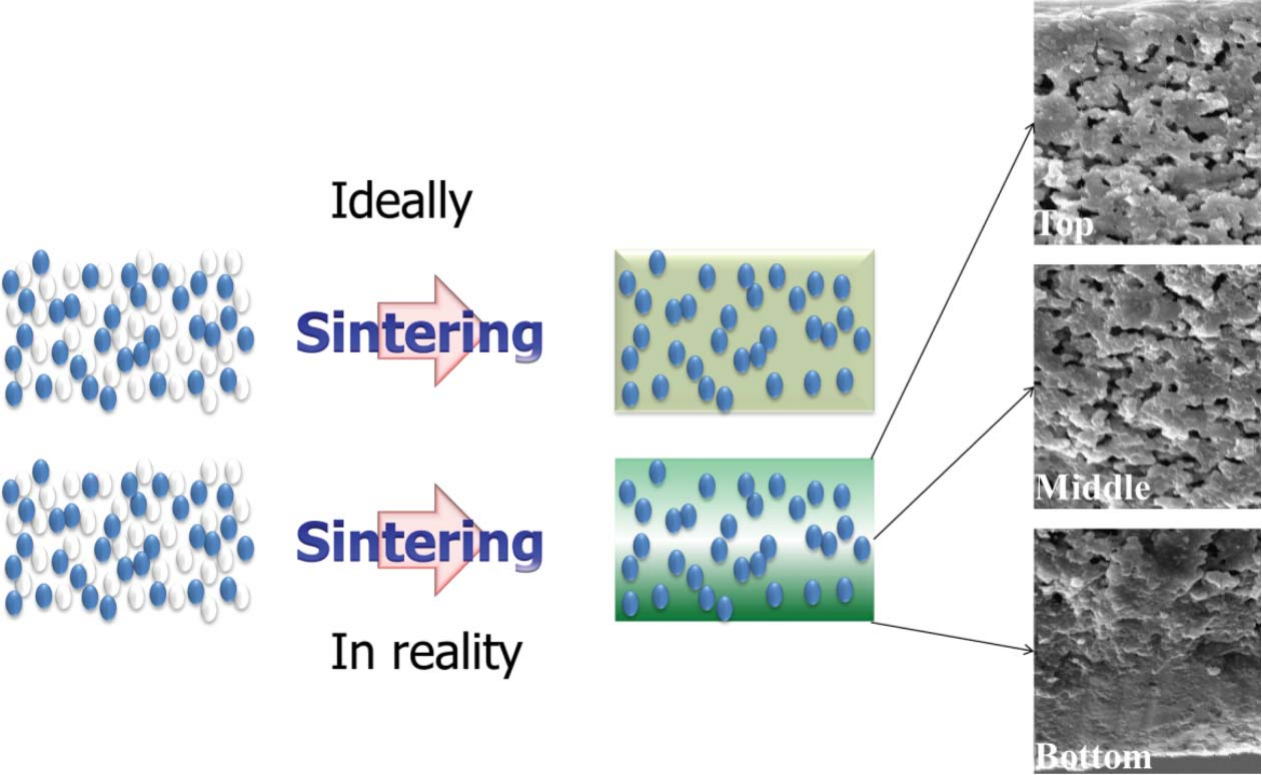
Glass spreading: expected vs. in reality.

The glass region which covered the surface of specimens was identified as shown in Figure 7. This is thought to accord with the result of Figure 6, as the specimen was polished and the dielectric constant was measured. As the covering dense glass region was removed, it is thought that the lower dielectric constant was measured after polishing.

It is expected that embedding passive components in the LTCC substrate without mismatch between different materials would make the mobile health care system more functional in less volume.

## Conclusions

Fillers with high dielectric constants were mixed with common glass and then co-fired. It was expected that fired LTCC material would show relatively high dielectric constants. When 1.3MgO-TiO_2_ with a volumetric ratio of 30% was mixed with Ca–Al–Si–O glass, the measured dielectric constant was 7.9, the quality factor was 3708. With 45 volumetric percent of cordierite, the dielectric constant was 5 and the quality factor was 1052.

CaTiO_3_ filler powders have very fine particle sizes, and this requires low viscosity glass. Low viscosity glass, however, sank out during firing to make much vacant spaces. It is thought that adequate dielectric constants with common glass in LTCC could be obtained with relatively coarse particles.
